# Bis(3-carbamoylpyridin-1-ium) phosphite mono­hydrate

**DOI:** 10.1107/S2056989018011192

**Published:** 2018-08-21

**Authors:** Jan Fábry

**Affiliations:** a Institute of Physics of the Czech Academy of Sciences, Na Slovance 2, 182 21 Praha 8, Czech Republic

**Keywords:** crystal structure, hydrogen bonding, phosphite

## Abstract

Two of the constituent mol­ecules in the title structure, 2C_6_H_7_N_2_O^+^·HPO_3_^2−^·H_2_O, *i.e.* the phosphite anion and the water mol­ecule, are situated on a symmetry plane. The mol­ecules are held together by moderate N—H⋯O and O—H⋯N, and weak O—H⋯O and C—H⋯O_carbon­yl_ hydrogen bonds in which the amide and secondary amine groups, and the water molecules are involved. The structural features are usual, among them the H atom bonded to the P atom avoids hydrogen bonding.

## Abstract 2

Two of the constituent mol­ecules in bis­(3-carbamoylpyridin-1-ium) phosphite monohydrate, *i.e.* the phosphite anion and the water mol­ecule, are situated on the symmetry plane. The mol­ecules are held together by moderate N—H⋯O and O—H⋯N, and weak O—H⋯O and C—H⋯O_carbon­yl_ hydrogen bonds in which the primary and secondary amine and water H atoms are involved. The H atom directly bonded to the P atom avoids hydrogen bonding, as usual.

## Chemical context   

Nicotinamide (pyridine-2-carboxamide) is a biologically important mol­ecule, being the active part of vitamin B3 and nicotinamide adenine dinucleotide (NAD) (*e.g.* Wald, 1991 ▸; Williamson *et al.*, 1967 ▸).
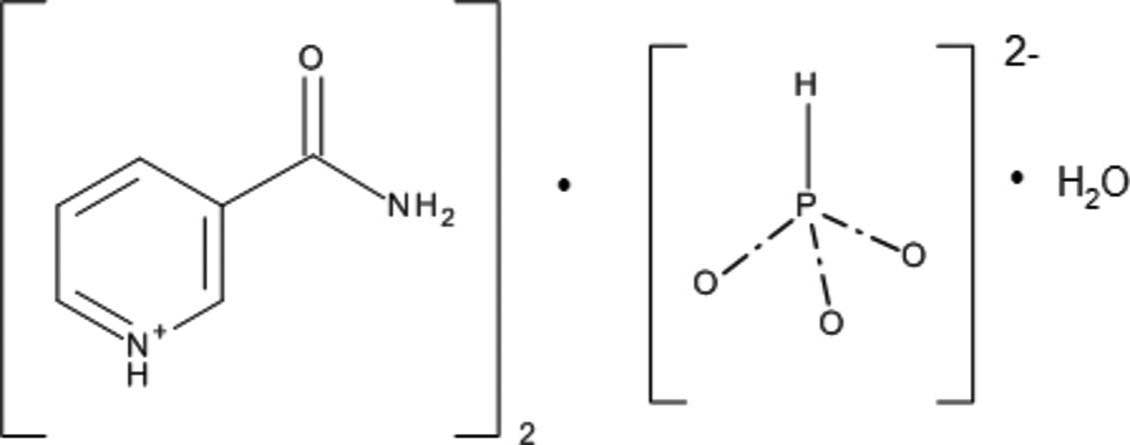


However, inter­est in the preparation of the title hydrated salt was called for with respect to an investigation of the configuration of the –NH_2_ group and its dependence on its environment.

It was hoped that 3-carbamoyl­pyridine (nicotinamide) would make a salt or a co-crystal with phospho­rous acid, H_3_PO_3_. It is difficult to predict which of these two forms would be prefererred, because of a small difference of Δp*K*_a_ = p*K*_a_(base) − p*K*_a_(acid) (Childs *et al.*, 2007 ▸). [The p*K*_a_ values for 3-carbamoyl­pyridine and H_3_PO_3_ are 3.3 and 1.3 (first degree), respectively (CRC Handbook, 2009 ▸).]

## Structural commentary   

**Figure 1:**
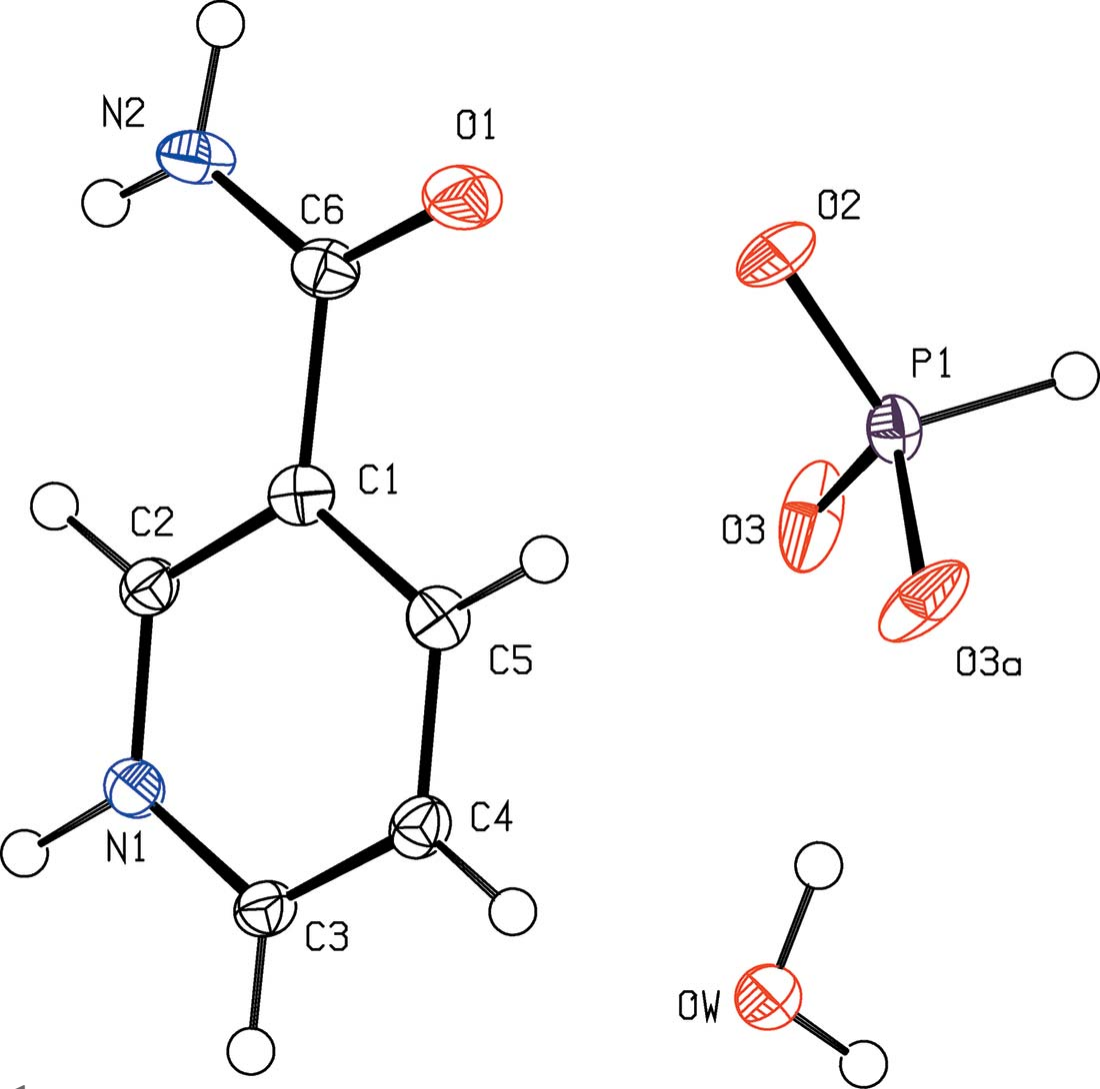
The title mol­ecule, with anisotropic atomic displacement ellipsoids shown at the 50% probability level (*PLATON*; Spek, 2009 ▸).

**Figure 2:**
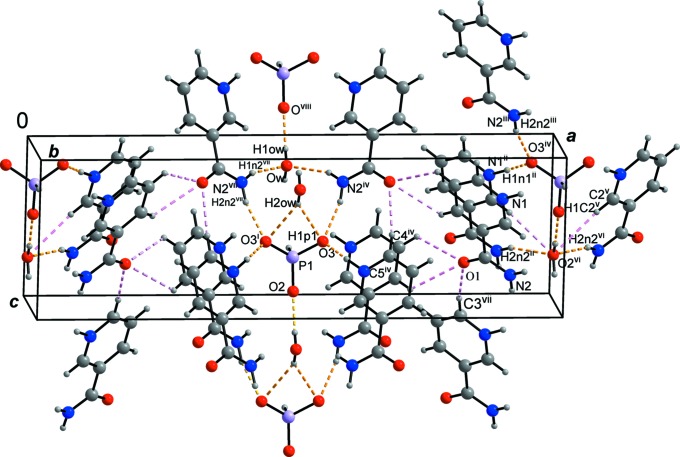
View of the title structure. C, H, N, O and P atoms are represented by gray, small gray, blue, red and violet circles, respectively. [Symmetry codes: (i) −*x* + 1, *y*, *z*; (ii) *x*, *y* − 1, *z*; (iii) *x*, *y* − 1, *z*; (iv) −*x* + 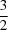, −*y* + 1, *z* − 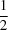; (v) −*x* + 2, *y* − 1, *z*; (vi) −*x* + 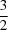, −*y* + 1, *z* + 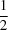; (vii) *x* − 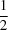, −*y* + 1, *z* − 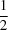; (viii) *x*, *y* − 1, *z* − 1.] The hydrogen bonds are shown as yellow dashed lines (*DIAMOND*; Brandenburg & Putz, 2005 ▸).

The title molecules are shown in Fig. 1 ▸. The resulting structure turned out to be a monohydrated salt. Table 1 ▸ lists the hydrogen bonds, which are shown in Fig. 2 ▸. The secondary amine hydrogen H1*n*1 is involved in the strongest hydrogen bond present in the structure (N1—H1*n*1⋯O3^i^). Its parameters indicate that this hydrogen bond is situated on the boundary between strong and moderate hydrogen bonds (Gilli & Gilli, 2009 ▸). The amide hydrogen H1*n*2 is donated to the water oxygen, while H2*n*2 is donated to atom O3 of the phosphite anion. Atom O2 is an acceptor of water hydrogen H1*ow*. Water hydrogen H2*ow* is donated to a pair of O3 atoms. The carbonyl oxygen O1 is an acceptor of two weak C—H⋯O hydrogen bonds, namely C3—H1*c*3⋯O1^ii^ and C4—H1*c*4⋯O1^iii^. The water oxygen atom is also an acceptor of hydrogen H1*c*2 (see Table 1 ▸).

**Figure 3:**
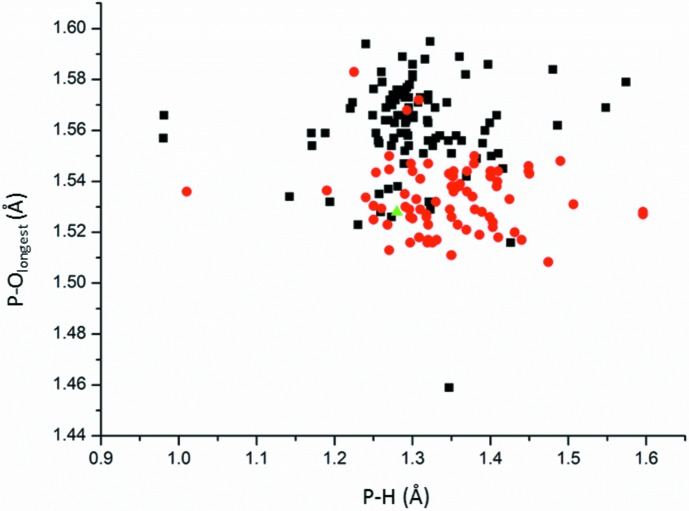
The dependence of the longest P—O distance (Å) on the P—H distance (Å) in hydrogen phosphites (red circles); phosphites are represented by black squares and the title phosphite structure by a green triangle.

Phosphite and fluoro­phospho­nate, as well hydrogen phosphite and hydrogen fluoro­phospho­nate, are similar mol­ecules. Either mol­ecule can be involved, not only in isostructural compounds, but even in mixed crystals (Fábry *et al.*, 2012 ▸). Similarity regarding not only the shape of the mol­ecules but also the avoidance both of P-bonded fluorines and hydrogens of involvement in strong or moderate hydrogen bonds (Matulková *et al.*, 2017 ▸). The latter article shows a plot of the dependence of P—F distance on the longest P—O distance in flouro­phospho­nate and hydrogen fluoro­phospho­nate mol­ecules. The P—F distance tends to be longer in [FPO_3_]^2−^ than in [HFPO_3_]^−^. Fig. 3 ▸ shows a similar plot for the phosphites and hydrogen phosphites between both mol­ecules despite the larger spread of P—H distances in phosphite mol­ecules because of the lower accuracy of the H-atom determinations by X-ray diffraction experiments. The reason why the P—H bond tends to be longer follows from the conservation of the overall bond valence sum of the central P^5+^ or P^3+^ atom. It is worth pointing out that the tabulated value of the bond valence parameter for the P—H bond seems to yield too high values. For example, for the important values of the P—H distances, *i.e.* 1.28, 1.33 and 1.37 Å (*cf.* Fig. 3 ▸), the bond valences (Brese & O’Keeffe, 1991 ▸) are 1.42, 1.24 and 1.11, respectively. The P—H bond valence parameters are going to be checked as part of future work.

The C—NH_2_ group tends to be fairly planar for short C—N bonds (Fábry *et al.*, 2014 ▸). In agreement with a short C—N bond length [C6—N2 = 1.3232 (18) Å] in the title structure, the best plane through C6/N2/H1*n*2/H2*n*2 reveals a maximum deviation of about 0.05 (2) Å for each hydrogen, while ξ^2^ = 12.6.

## Supra­molecular features   

In the crystal, the most important graph-set motif (Etter *et al.*, 1990 ▸) present is an 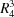
(10) ring motif, which is composed of atoms P1—O3⋯H2*n*2^iv^—N2^iv^—H1*n*2^iv^⋯O*w*⋯H1*n*2^vii^—N2^vii^—H2*n*2^vii^⋯O3^i^ (Table 1 ▸ and Fig. 2 ▸; symmetry codes are given in the figure cation). The phosphite anion and the water mol­ecule are linked by O_water_—H⋯O_phosphite_ hydrogen bonds, forming chains propagating along [001]. The cations are linked to these chains *via* N—H⋯O hydrogen bonds, forming layers parallel to the *bc* plane, as shown in Fig. 2 ▸. The layers are linked by C—H⋯O hydrogen bonds, resulting in the formation of a supra­molecular three-dimensional structure.

## Database survey   

The applied crystallographic databases were the Cambridge Crystallographic Database (Version 5.39, with updates to May 2018; Groom *et al.*, 2016 ▸) and the Inorganic Crystal Structure Database (June 2018; ICSD, 2018 ▸). The search was carried out for all phosphites or hydrogen phosphites with a cation of one kind.

## Synthesis and crystallization   

The title structure was prepared by slow evaporation of a water solution (18 ml) of equimolar amounts of nicotinamide (1.49 g) and phospho­rous acid (1 g). Colourless crystals were isolated after two months.

## Refinement   

Crystal data, data collection and structure refinement details are summarized in Table 2 ▸. All the H atoms were discernible in the difference electron-density map. The aryl H atoms were constrained by the constraints C—H = 0.95 Å and *U*_iso_(H) = 1.2*U*_eq_(C). Water hydrogen H2*ow* was refined freely, while H1*ow* was restrained with a distance restraint of 0.84 Å with elasticity 0.02 Å (Müller, 2009 ▸), and with *U*_iso_(H) = 1.5*U*_eq_(O). The hydrogens of the primary amine N2 group and the secondary amine N1 group were constrained by *U*_iso_(H) = 1.2*U*_eq_(N). The P—H hydrogen was refined isotropically. Three reflections, *i.e.* 95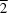, 10,5,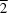 and 11,5,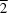, were discarded from the refinement because |*I*(obs) − *I*(calc)|/σ(*I*) > 20.

Since the phosphite oxygens revealed large displacement ellipsoids, the anharmonic displacement parameters upto the fourth grade were included for atoms P1, O2 and O3. (The refinement with the harmonic approximation resulted in *R*_obs_ = 0.0242, *Rw*_obs_ = 0.0773, *R*_all_ = 0.0243, *Rw*_all_ = 0.0774 and *S* = 3.13, with number of parameters = 127. With application of anharmonic approximation, *R*_obs_ = 0.0188, *Rw*_obs_ = 0.0535, *R*_all_ = 0.0188, *Rw*_all_ = 0.0535 and *S* = 2.21, with number of parameters = 181. The respective values of the third- and fourth-order components of the displacement tensor are given in the CIF.)

Refinement with the assumption of the presence of inversion twinning resulted in a Flack parameter of 0.012 (13) (726 Friedel pairs used in the refinement). Therefore, the crystal was considered as single-domained in the final stage of the refiement, the results of which are presented here.

## Supplementary Material

Crystal structure: contains datablock(s) global, I. DOI: 10.1107/S2056989018011192/eb2010sup1.cif

Structure factors: contains datablock(s) I. DOI: 10.1107/S2056989018011192/eb2010Isup2.hkl

Supporting information file. DOI: 10.1107/S2056989018011192/eb2010Isup3.smi

Supporting information file. DOI: 10.1107/S2056989018011192/eb2010Isup4.cml

CCDC reference: 1860376

Additional supporting information:  crystallographic information; 3D view; checkCIF report

## Figures and Tables

**Table 1:** Hydrogen-bond geometry (Å, °)

*D*—H⋯*A*	*D*—H	H⋯*A*	*D*⋯*A*	*D*—H⋯*A*
C2—H1*c*2⋯O*w* ^i^	0.95	2.64	3.5777 (14)	168
C3—H1*c*3⋯O1^ii^	0.95	2.56	3.4790 (17)	164
C4—H1*c*4⋯O1^iii^	0.95	2.56	3.1948 (13)	125
N1—H1*n*1⋯O3^iv^	1.053 (15)	1.455 (15)	2.508 (3)	178.1 (14)
N2—H1*n*2⋯O*w* ^i^	0.849 (16)	2.140 (16)	2.9513 (13)	159.8 (18)
N2—H2*n*2⋯O3^v^	0.889 (18)	1.955 (18)	2.823 (3)	165.2 (15)
O*w*—H1*ow*⋯O2^vi^	0.84 (2)	1.82 (2)	2.657 (4)	172 (2)
O*w*—H2*ow*⋯O3	0.96 (3)	2.42 (2)	3.263 (3)	146.8 (9)
O*w*—H2*ow*⋯O3^vii^	0.96 (3)	2.42 (2)	3.263 (3)	146.8 (9)

Symmetry codes: (i) 
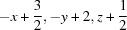
; (ii) 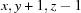; (iii) 
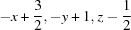
; (iv) 
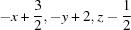
; (v) 
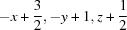
; (vi) 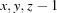; (vii) 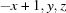.

**Table 2:** Experimental details

Crystal data	
Chemical formula	2C_6_H_7_N_2_O^+^·HPO_3_^2−^·H_2_O
*M*_r_	344.3
Crystal system, space group	Orthorhombic, *P* *m* *n*2_1_
Temperature (K)	95
*a*, *b*, *c* (Å)	22.9297 (4), 4.5910 (1), 7.0900 (1)
*V* (Å^3^)	746.37 (2)
*Z*	2
Radiation type	Cu *K*α
μ (mm^−1^)	2.01
Crystal size (mm)	0.45 × 0.16 × 0.06

Data collection	
Diffractometer	Rigaku OD SuperNova Dual source diffractometer with an AtlasS2 detector
Absorption correction	Multi-scan (*CrysAlis PRO*; Rigaku OD, 2017 ▸)
*T*_min_, *T*_max_	0.599, 0.831
No. of measured, independent and observed [*I* > 3σ(*I*)] reflections	10796, 1592, 1588
*R*_int_	0.021
(sin θ/λ)_max_ (Å^−1^)	0.630

Refinement	
*R*[*F* > 3σ(*F*)], *wR*(*F*), *S*	0.019, 0.054, 2.21
No. of reflections	1592
No. of parameters	181
No. of restraints	1
H-atom treatment	H atoms treated by a mixture of independent and constrained refinement
Δρ_max_, Δρ_min_ (e Å^−3^)	0.14, −0.15
Absolute structure	Since the Flack parameter turned out to equal to 0.012(13) in the final stage of refinement it was set to 0. 726 Friedel pairs used in the refinement.
Absolute structure parameter	0.0

Computer programs: *CrysAlis PRO* (Rigaku OD, 2017 ▸), *SIR2014* (Burla *et al.*, 2015 ▸), *JANA2006* (Petříček *et al.*, 2014 ▸), *PLATON* (Spek, 2009 ▸) and *DIAMOND* (Brandenburg & Putz, 2005 ▸); extinction correction according to Becker & Coppens (1974 ▸).

## supplementary crystallographic information

### Crystal data

**Table:**

2C_6_H_7_N_2_O^+^·HPO_3_^2^^−^·H_2_O	*F*(000) = 360
*M**_r_* = 344.3	*D*_x_ = 1.532 Mg m^−^^3^
Orthorhombic, *P**m**n*2_1_	Cu *K*α radiation, λ = 1.54184 Å
Hall symbol: P -2x;-2yac;2zac	Cell parameters from 9530 reflections
*a* = 22.9297 (4) Å	θ = 6.5–75.9°
*b* = 4.5910 (1) Å	µ = 2.01 mm^−^^1^
*c* = 7.0900 (1) Å	*T* = 95 K
*V* = 746.37 (2) Å^3^	Plate, colourless
*Z* = 2	0.45 × 0.16 × 0.06 mm

### Data collection

**Table:**

Rigaku OD SuperNova Dual source diffractometer with an AtlasS2 detector	1592 independent reflections
Radiation source: micro-focus sealed X-ray tube, SuperNova (Cu) X-ray Source	1588 reflections with *I* > 3σ(*I*)
Mirror monochromator	*R*_int_ = 0.021
Detector resolution: 5.2027 pixels mm^-1^	θ_max_ = 76.3°, θ_min_ = 3.9°
ω/ scans	*h* = −28→28
Absorption correction: multi-scan (CrysAlis PRO; Rigaku OD, 2017)	*k* = −5→5
*T*_min_ = 0.599, *T*_max_ = 0.831	*l* = −8→8
10796 measured reflections	

### Refinement

**Table:**

Refinement on *F*^2^	Weighting scheme based on measured s.u.'s *w* = 1/(σ^2^(*I*) + 0.0004*I*^2^)
*R*[*F* > 3σ(*F*)] = 0.019	(Δ/σ)_max_ = 0.035
*wR*(*F*) = 0.054	Δρ_max_ = 0.14 e Å^−^^3^
*S* = 2.21	Δρ_min_ = −0.15 e Å^−^^3^
1592 reflections	Extinction correction: B-C type 1 Lorentzian isotropic (Becker & Coppens, 1974)
181 parameters	Extinction coefficient: 910 (160)
1 restraint	Absolute structure: Since the Flack parameter turned out to equal to 0.012(13) in the final stage of refinement it was set to 0. 726 Friedel pairs used in the refinement.
22 constraints	Absolute structure parameter: 0.0
H atoms treated by a mixture of independent and constrained refinement	

### Special details

**Table:**

Refinement. This part differs from the original article by Thanigaimani *et al.* (2006). It also differs from the refinement by Thanigaimani *et al.* (2006) by a different threshold for the consideration of the observed diffractions: F^2^ > 3sigma(F^2^) has been used as criterion for observed diffractions by *JANA*2006 which was used for the calculation of the corrected structural model.Three diffractions 9 5 -2, 10 5 -2, 11 5 -2 were discarded from the refinement because |I(obs)-I(calc)|/σ(I) > 20. Since the Flack parameter turned out to equal to 0.012 (13) in the final stage of refinement it was set to 0. 726 Friedel pairs used in the refinement.

### Fractional atomic coordinates and isotropic or equivalent isotropic displacement parameters (Å^2^)

**Table:**

	*x*	*y*	*z*	*U*_iso_*/*U*_eq_	
C1	0.84788 (5)	0.8441 (2)	0.47478 (18)	0.0157 (3)	
C2	0.88464 (4)	1.0560 (2)	0.40131 (18)	0.0159 (3)	
H1c2	0.916985	1.121048	0.473657	0.0191*	
N1	0.87528 (4)	1.1699 (2)	0.23035 (17)	0.0168 (2)	
H1n1	0.9056 (7)	1.320 (3)	0.175 (2)	0.0202*	
C3	0.82992 (5)	1.0852 (2)	0.1232 (2)	0.0169 (3)	
H1c3	0.824169	1.170176	0.002456	0.0202*	
C4	0.79162 (5)	0.8751 (2)	0.1879 (2)	0.0178 (3)	
H1c4	0.759552	0.814668	0.112553	0.0213*	
C5	0.80087 (4)	0.7536 (2)	0.36522 (18)	0.0168 (3)	
H1c5	0.77504	0.608615	0.411623	0.0201*	
C6	0.85727 (5)	0.7074 (3)	0.66613 (19)	0.0173 (3)	
O1	0.82199 (4)	0.52392 (19)	0.72395 (16)	0.0271 (2)	
N2	0.90344 (4)	0.7930 (2)	0.76358 (18)	0.0202 (3)	
H1n2	0.9281 (7)	0.917 (3)	0.724 (3)	0.0243*	
H2n2	0.9105 (7)	0.698 (4)	0.871 (3)	0.0243*	
P1	0.5	0.3758 (3)	0.7085 (2)	0.0181 (6)	
H1p1	0.5	0.092 (5)	0.698 (4)	0.034 (6)*	
O2	0.5	0.4732 (8)	0.9100 (6)	0.0287 (14)	
O3	0.55414 (11)	0.4668 (5)	0.5974 (3)	0.0350 (8)	
Ow	0.5	0.7861 (2)	0.22531 (19)	0.0219 (3)	
H1ow	0.5	0.672 (5)	0.132 (3)	0.0328*	
H2ow	0.5	0.658 (6)	0.332 (4)	0.0328*	

### Atomic displacement parameters (Å^2^)

**Table:**

	*U*^11^	*U*^22^	*U*^33^	*U*^12^	*U*^13^	*U*^23^
C1	0.0143 (4)	0.0170 (5)	0.0159 (5)	0.0034 (3)	0.0021 (4)	−0.0004 (4)
C2	0.0157 (4)	0.0167 (5)	0.0152 (5)	0.0009 (4)	−0.0018 (4)	−0.0008 (4)
N1	0.0174 (4)	0.0158 (4)	0.0172 (4)	0.0004 (3)	−0.0006 (4)	0.0018 (4)
C3	0.0183 (4)	0.0170 (5)	0.0153 (5)	0.0020 (4)	−0.0026 (4)	0.0000 (4)
C4	0.0163 (4)	0.0189 (5)	0.0182 (5)	0.0008 (3)	−0.0032 (4)	−0.0018 (4)
C5	0.0142 (4)	0.0177 (5)	0.0184 (5)	−0.0001 (4)	0.0019 (3)	−0.0001 (4)
C6	0.0176 (5)	0.0183 (5)	0.0161 (5)	0.0013 (4)	0.0026 (3)	0.0014 (4)
O1	0.0275 (4)	0.0331 (4)	0.0207 (4)	−0.0115 (3)	−0.0015 (4)	0.0082 (4)
N2	0.0179 (4)	0.0265 (5)	0.0162 (4)	−0.0015 (4)	−0.0011 (3)	0.0072 (4)
P1	0.0192 (11)	0.0147 (7)	0.0205 (12)	0	0	−0.0018 (8)
O2	0.038 (3)	0.040 (2)	0.008 (2)	0	0	−0.0080 (19)
O3	0.0349 (14)	0.0382 (15)	0.0318 (15)	−0.0234 (11)	0.0241 (13)	−0.0182 (12)
Ow	0.0283 (5)	0.0185 (5)	0.0188 (5)	0	0	−0.0010 (5)

### Geometric parameters (Å, º)

**Table:**

C1—C2	1.3884 (15)	C5—H1c5	0.95
C1—C5	1.3920 (16)	C6—O1	1.2379 (15)
C1—C6	1.5103 (18)	C6—N2	1.3237 (16)
C2—H1c2	0.95	N2—H1n2	0.849 (16)
C2—N1	1.3375 (17)	N2—H2n2	0.889 (18)
N1—H1n1	1.053 (15)	H1n2—H2n2	1.50 (2)
N1—C3	1.3455 (16)	P1—H1p1	1.30 (3)
H1n1—O3^i^	1.455 (15)	P1—O2	1.497 (4)
C3—H1c3	0.9501	P1—O3	1.528 (3)
C3—C4	1.3827 (15)	P1—O3^ii^	1.528 (3)
C4—H1c4	0.95	Ow—H1ow	0.84 (2)
C4—C5	1.3917 (18)	Ow—H2ow	0.96 (3)
			
C2—C1—C5	118.02 (11)	C1—C5—H1c5	119.91
C2—C1—C6	122.79 (10)	C4—C5—H1c5	119.91
C5—C1—C6	119.19 (10)	C1—C6—O1	119.15 (11)
C1—C2—H1c2	119.45	C1—C6—N2	117.36 (10)
C1—C2—N1	121.10 (10)	O1—C6—N2	123.49 (13)
H1c2—C2—N1	119.45	C6—N2—H1n2	124.0 (12)
C2—N1—H1n1	119.1 (9)	C6—N2—H2n2	116.4 (11)
C2—N1—C3	121.51 (10)	H1n2—N2—H2n2	119.2 (16)
H1n1—N1—C3	119.3 (9)	H1p1—P1—O2	110.6 (13)
N1—H1n1—O3^i^	178.1 (14)	H1p1—P1—O3	104.1 (7)
N1—C3—H1c3	119.82	H1p1—P1—O3^ii^	104.1 (7)
N1—C3—C4	120.37 (12)	O2—P1—O3	114.21 (12)
H1c3—C3—C4	119.82	O2—P1—O3^ii^	114.21 (12)
C3—C4—H1c4	120.58	O3—P1—O3^ii^	108.64 (15)
C3—C4—C5	118.82 (11)	H1n1^iii^—O3—P1	120.3 (6)
H1c4—C4—C5	120.59	H1ow—Ow—H2ow	104 (2)
C1—C5—C4	120.18 (10)		

Symmetry codes: (i) −*x*+3/2, −*y*+2, *z*−1/2; (ii) −*x*+1, *y*, *z*; (iii) −*x*+3/2, −*y*+2, *z*+1/2.

### Hydrogen-bond geometry (Å, º)

**Table:**

*D*—H···*A*	*D*—H	H···*A*	*D*···*A*	*D*—H···*A*
C2—H1*c*2···O*w*^iii^	0.95	2.64	3.5777 (14)	167.77
C3—H1*c*3···O1^iv^	0.95	2.56	3.4790 (17)	163.62
C4—H1*c*4···O1^v^	0.95	2.56	3.1948 (13)	124.74
N1—H1*n*1···O3^i^	1.053 (15)	1.455 (15)	2.508 (3)	178.1 (14)
N2—H1*n*2···O*w*^iii^	0.849 (16)	2.140 (16)	2.9513 (13)	159.8 (18)
N2—H2*n*2···O3^vi^	0.889 (18)	1.955 (18)	2.823 (3)	165.2 (15)
O*w*—H1*ow*···O2^vii^	0.84 (2)	1.82 (2)	2.657 (4)	172 (2)
O*w*—H2*ow*···O3	0.96 (3)	2.42 (2)	3.263 (3)	146.8 (9)
O*w*—H2*ow*···O3^ii^	0.96 (3)	2.42 (2)	3.263 (3)	146.8 (9)

Symmetry codes: (i) −*x*+3/2, −*y*+2, *z*−1/2; (ii) −*x*+1, *y*, *z*; (iii) −*x*+3/2, −*y*+2, *z*+1/2; (iv) *x*, *y*+1, *z*−1; (v) −*x*+3/2, −*y*+1, *z*−1/2; (vi) −*x*+3/2, −*y*+1, *z*+1/2; (vii) *x*, *y*, *z*−1.
